# “Help in a Heartbeat?”: A Systematic Evaluation of Mobile Health Applications (Apps) for Coronary Heart Disease

**DOI:** 10.3390/ijerph181910323

**Published:** 2021-09-30

**Authors:** Chiara Mack, Yannik Terhorst, Mirjam Stephan, Harald Baumeister, Michael Stach, Eva-Maria Messner, Jürgen Bengel, Lasse B. Sander

**Affiliations:** 1 Department of Rehabilitation Psychology and Psychotherapy, Institute of Psychology, Albert-Ludwigs-University of Freiburg, 79085 Freiburg, Germany; chiara.mack.96@gmail.com (C.M.); miri.stephan@mail.de (M.S.); bengel@psychologie.uni-freiburg.de (J.B.); 2 Department of Clinical Psychology and Psychotherapy, Institute of Psychology and Education, Ulm University, 89040 Ulm, Germany; yannik.terhorst@uni-ulm.de (Y.T.); harald.baumeister@uni-ulm.de (H.B.); eva-maria.messner@uni-ulm.de (E.-M.M.); 3 Institute of Databases and Information Systems, Ulm University, 89040 Ulm, Germany; michael.stach@uni-ulm.de

**Keywords:** coronary heart disease (CHD), apps, mobile health, eHealth, systematic evaluation

## Abstract

For patients with coronary heart disease (CHD) lifestyle changes and disease management are key aspects of treatment that could be facilitated by mobile health applications (MHA). However, the quality and functions of MHA for CHD are largely unknown, since reviews are missing. Therefore, this study assessed the general characteristics, quality, and functions of MHA for CHD. Hereby, the Google Play and Apple App stores were systematically searched using a web crawler. The general characteristics and quality of MHA were rated with the Mobile Application Rating Scale (MARS) by two independent raters. From 3078 identified MHA, 38 met the pre-defined criteria and were included in the assessment. Most MHA were affiliated with commercial companies (52.63%) and lacked an evidence-base. An overall average quality of MHA (*M* = 3.38, *SD* = 0.36) was found with deficiencies in information quality and engagement. The most common functions were provision of information and CHD risk score calculators. Further functions included reminders (e.g., for medication or exercises), feedback, and health management support. Most MHA (81.58%) had one or two functions and MHA with more features had mostly higher MARS ratings. In summary, this review demonstrated that a number of potentially helpful MHA for patients with CHD are commercially available. However, there is a lack of scientific evidence documenting their usability and clinical potential. Since it is difficult for patients and healthcare providers to find suitable and high-quality MHA, databases with professionally reviewed MHA are required.

## 1. Introduction

Cardiovascular diseases and especially coronary heart diseases (CHD) are one of the leading causes of death worldwide [1,2]. According to the global burden of disease study 17.8 million people died from cardiovascular diseases in 2017 [1]. According to the heart disease and stroke statistics the prevalence of CHD in the US ranges from 5.3% for female adults to 7.4% for male adults [3].

CHD and common complications like arrhythmia, myocardial infarction, and heart failure have a significant negative impact on the affected person’s health, leading to high mortality and healthcare costs [4,5,6]. In addition, certain mental and physical conditions and factors are associated with CHD, including depression, cigarette smoking, hypertension, and obesity [7,8,9,10].

Disease management and behavior change including lifestyle changes are key aspects of CHD care but often not adequately and enduringly considered in care settings [11]. The large number of risk and lifestyle factors render the prevention and self-management of CHD extensive and complex for patients [11,12]. Therefore, means of promoting disease management and lifestyle changes as well as information are necessary to improve prevention and conventional treatment of CHD [11,13,14]. Mobile health applications (MHA) are discussed to contribute in overcoming this gap in treatment by fostering CHD management [13,15]. First, MHA may support daily monitoring of activities and symptoms [16]. Second, adherence to treatment and lifestyle changes can be increased by self-tracking, feedback, and reminder functions of MHA [16,17]. Third, MHA are accessible at all times and at relatively little costs [18] making MHA a scalable solution to provide general information about CHD, symptoms, and specific lifestyle modifications [19,20]. Fourth, MHA can increase patients’ perception to play an active role in their own healthcare and hereby foster self-sufficiency, disease management, and patient autonomy [16,18,21].

However, high-quality applications with suitable content are required, while the quality of MHA is largely unknown due to an intransparent MHA market and a lack of methodologically sound quality assessments [16,22]. Previous studies focused on other cardiological conditions examining the quality of MHA for heart failure [23,24], atrial fibrillation [25], and blood pressure [26]. Hereby the quality of MHA was reported as mostly acceptable [24] or mainly poor [23,26]. This is particularly alarming because MHA can also be harmful [19,22]. Risks and constraints regarding MHA concern data security, privacy, and confidentiality, since missing privacy policies and information transfer to third parties have been observed [22,27]. Furthermore, possible misinformation poses potential risks to users and the sheer number of MHA may lead to consumer confusion [11,19,28,29,30]. No evaluation of MHA specifically for CHD was found [16].

Therefore, in this study we systematically searched for and conducted a standardized evaluation of MHA for CHD which are available in commercial app stores. Hereby we addressed the following research questions:

1. What is the quality of CHD applications in European commercial app stores in regard to engagement, functionality, aesthetics, and information quality in general?
2. What functions are employed in CHD applications?

## 2. Materials and Methods

### 2.1. App Search Strategy

With an automated search engine (web crawler) of the ‘Mobile Health Application Database’ (MHAD) [31] the Google Play store and Apple App store were systematically searched for MHA. Search terms to identify CHD applications included ‘coronary heart disease’, ‘coronary artery disease’, ‘ischemic heart disease’, and ‘heart disease’ in English and German. A list of all search terms is included in Appendix A. The searches were conducted between December 2020 and February 2021. Duplicates were automatically removed. For the assessment, MHA from the Google Play store were installed on an Honor 6X (BLL-L22) and apps from the Apple App store on an iPad Pro A1652.

### 2.2. Inclusion Criteria and Process

The identified MHA were examined for eligibility in a two-step procedure. In the first step, the title and app description were screened and the inclusion criteria for the download of MHA were applied. Apps were downloaded if (a) in the app title or description the subject of coronary heart disease was stated, (b) the app was developed for patients with CHD, persons at risk, or otherwise affected individuals, (c) the MHA was available in German or English language, and (d) download was possible.

In a second step, the identified apps were downloaded and the criteria for inclusion in the evaluation were examined within the app. MHA were included if (a) CHD was focused, a CHD-specific section was included, or the app description stated its use for CHD, (b) no other specific information (such as login/ access data) was required for usage of the app, (c) the application was functional, and d) there were no further technical reasons to eliminate the MHA. Technical malfunctions were tested on two devices.

### 2.3. Data Extraction, Evaluation Criteria, and Instruments

Two independent raters (master’s degree students in psychology C.M. and M.S., under supervision of a licensed psychotherapist L.B.S.) conducted the acquisition and rating of all included MHA by applying the Mobile Application Rating Scale (MARS) in the German version [32,33,34]. For all sections of the MARS a good to excellent internal consistency (Omega = 0.793 to 0.904), an overall excellent internal consistency (Omega = 0.929) and a good intra-class correlation (ICC = 0.816, 95% CI: 0.810 to 0.822) were shown [34]. Therefore, with the MARS the quality of MHA can be assessed reliably [34]. The MARS contains a section for classification and for quality rating as well as three additional subscales.

To prepare for the app rating with the MARS, a free online tutorial provided by the developers of the German MARS version was viewed. For the rating, each app was tested by trying out all features. To check the agreement between the raters, the interrater reliability (IRR) was calculated. Here, the intra-class correlation (ICC) needs to be ≥ 0.75 to indicate a sufficient agreement [35]. In case of an ICC below 0.75 the supervisor (L.B.S.) was consulted.

### 2.4. General Characteristics of MHA

For this study the MARS classification section was adapted to include the following general characteristics: (1) app name, (2) platform (Android, iOS), (3) affiliation, (4) price, (5) embedment in therapy, (6) user star rating, (7) number of user ratings, (8) app store category, (9) methods, (10) technical aspects, and (11) security and privacy.

### 2.5. Quality Rating

For the quality rating with the MARS 19 items are rated on a five-point scale ranging from 1 (inadequate) to 5 (excellent). These items constitute the four dimensions: (A) engagement (five items: entertainment, interest, individual adaptability, interactivity, target group), (B) functionality (four items: performance, usability, navigation, gestural design), (C) aesthetics (three items: layout, graphics, visual appeal), and (D) information quality (seven items: accuracy of app description, goals, quality of information, quantity of information, quality of visual information, credibility, evidence base). To assess the evidence-base, for each MHA Google Scholar was searched for published studies.

### 2.6. Statistical Analysis

For each of these four dimensions, the mean score (*M*) and standard deviation (*SD*) were computed as well as a total mean quality score across all four objective dimensions [33]. The scores of both raters were averaged. Additionally, the three subjective subscales of the MARS: (E) therapeutic gain, (F) subjective quality, and (G) perceived impact were evaluated without effect on the overall mean score. Correlation analyses between the available user star ratings (one star to five stars) and the MARS total mean score as well as the objective dimensions were conducted if at least three ratings were available.

### 2.7. Assessment of Functions

Subsequently, the employed functions of the included MHA were assessed with a classification from the ‘Chances and Risks of Mobile Health Apps’ (CHARISMHA) study [36]. The classification is divided into six categories with one to five subcategories each. These are: provision of information (news, reference, learning material, player/viewer, broker), data acquisition, processing, and evaluation (decision support, calculator, meter, monitor, surveillance/tracker), administrative use (administration), calendar and appointment-related apps (diary, reminder, calendar), support (utility/aid, coach, health manager) and other (actuator, communicator, game, store, other). Hereby, for each MHA it was examined which functions are employed. Additionally, a correlation between the number of functions and the MARS total score was calculated.

## 3. Results

### 3.1. Search

**Figure 1:**
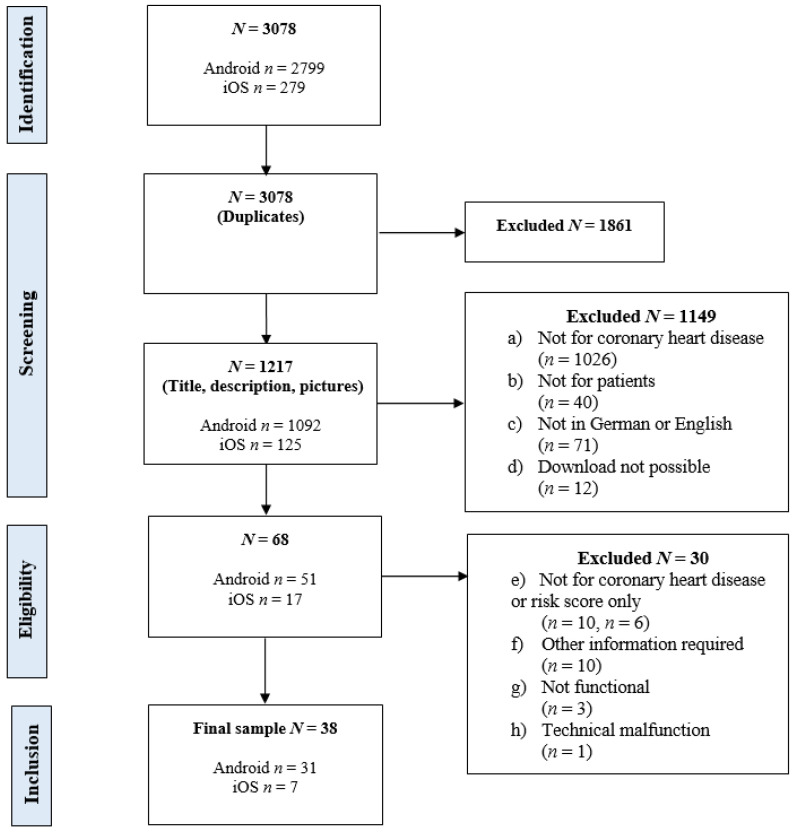
Flowchart of the app screening and inclusion process.

In Figure 1 the screening and inclusion process is illustrated. A total of 3078 apps were found through the web crawler. From 1217 apps without duplicates, 38 MHA (3.12%) were included in the evaluation. Of those, 30 apps (78.95%) were available on android, seven apps (18.42%) on iOS, and one app (2.63%) for both.

### 3.2. General Characteristics

The characteristics of included MHA are depicted in Table 1. The apps were affiliated with commercial companies (*n* = 20, 52.63%), non-governmental organizations (NGO; *n* = 2, 5.26%), universities (*n* = 2, 5.26%), and governments (*n* = 1, 2.63%). For 13 apps (34.21%) the affiliation was unknown. The basic version was free of cost for most apps (*n* = 34, 89.47%) and required payment for four apps (10.53%) with prices ranging from EUR 1.09 to EUR 3.69 (*M* = 2.57, *SD* = 1.08). In three apps (7.89%) an upgraded or extended pro version was available or in-app purchases were possible. No app was embedded in a treatment concept or had a certification to comply for example with the medical device regulation.

For 12 apps (31.58%) a user rating was available in the Google Play store and for one app (2.63%) in the Apple App store. The median user star rating in the Google Play store was 4.4 (*M* = 4.26, *SD* = 0.47) with five to 1276 ratings (*M* = 220.42, *SD* = 403.29) and the user star rating in the Apple App store was 1.0 with one rating (user ratings last updated on 4 April 2021). MHA were classified in eight app store categories: ‘Health & Fitness’ (*n* = 18, 47.37%), ‘Medical’ (*n* = 10, 26.32%), ‘Education’, ‘Books & Reference’ (*n* = 3, 7.89% each), ‘Lifestyle’, ‘Food & Drink’, ‘Entertainment’, and ‘Social Networking’ (*n* = 1, 2.63% each). In 19 apps (50.00%) internet was required for some or all functions and one app (2.63%) had an app community. Most common methods were information and education (*n* = 34, 89.47%), tips and advice (*n* = 25, 65.79%), and feedback (*n* = 16, 42.11%). For most apps a privacy policy (*n* = 26, 68.42%) and contact information (*n* = 33, 86.84%) was provided and in seven apps (18.42%) active consent was required. Login was necessary in six apps (15.79%) and a password protection in three apps (7.89%).

For one MHA (‘The Heart App’) the accuracy to detect acute coronary syndromes was examined in a diagnostic accuracy study [37]. Otherwise, no study or randomized controlled trial (RCT) was found.

### 3.3. Quality Rating of MHA

The MARS rating results for each included MHA are presented in Table 2. The total quality of included MHA was average, with *M* = 3.38 (SD = 0.36) and ranged from *M* = 2.50 to *M* = 4.22. Of the four objective dimensions, the highest-rated was functionality (*M* = 4.06, *SD* = 0.31), thereafter aesthetics (*M* = 3.62, *SD* = 0.47), followed by information quality (*M* = 3.18, *SD* = 0.43), and engagement (*M* = 2.64, *SD* = 0.55). For the additional subjective subscales, the means were lower, with *M* = 2.49 (*SD* = 0.35) for therapeutic gain, *M* = 2.45 (*SD* = 0.52) for subjective quality, and *M* = 1.90 (*SD* = 0.35) for perceived impact. The IRR for the rating of all MHA was excellent (2-way mixed ICC = 0.944, 95%-CI 0.935 to 0.952) and for no single app an ICC below 0.75 was evident. No significant correlations between user star ratings and MARS total mean score (*r* (10) = -0.52, *p* = 0.080) or the objective subscales (*r* (10) = 0.001–0.52, *p* > 0.05) were found. In addition, none of the apps that required payment for the basic version were among the ten highest-rated apps.

### 3.4. Functions of MHA

The number of functions of all MHA is presented in Appendix B. The most common function was provision of information, specifically reference (information texts about e.g., CHD, risk factors, or treatment) in 37 apps (97.37%) and a player/viewer (e.g., video or audio files) in six apps (15.79%). Hereof 28 MHA (73.68%) were primarily assigned to the category provision of information. Further functions were decision support (e.g., concerning foods or goals; *n* = 4, 10.53%), calculators such as CHD risk score or body mass index (BMI) calculators (*n* = 13, 34.21%) and monitor of activity (*n* = 1, 2.63%). Hereof six MHA (15.79%) were primarily categorized under data acquisition, processing, and evaluation. Other functions were diary (*n* = 1, 2.63%), reminder (e.g., for medication or workouts) and calendar functions (*n* = 3, 7.89% each), with no MHA primarily being a calendar and appointment-related app. Three MHA (7.89%) functioned as health managers, targeting goals regarding exercise, weight, and nutrition, and were categorized as support apps. Two MHA (5.26%) were communicators and of those, one app (2.63%) was primarily a social network.

The functions of the ten highest-rated apps are shown in Table 3 and a full table depicting all employed functions per MHA is included in Appendix C. In general, many MHA had one (*n* = 17, 44.74%) or two functions (*n* = 14, 36.84%) and in seven apps (18.42%) three or more functions were employed. Of those MHA with three or more functions, five apps (71.43%) were among the ten highest-rated apps. A significant positive correlation with a large effect size was found between the MARS total score and the number of employed functions (*r* (36) = 0.66, *p* < 0.001).

## 4. Discussion

This study is the first to systematically review MHA for CHD by assessing the general characteristics, quality, and functions of MHA in European app stores. The overall quality of apps for CHD, as assessed with the MARS, was average (*M* = 3.38, *SD* = 0.36). Here, the functionality and aesthetics of included apps were generally high while deficits in information quality and engagement were shown. This is in line with previous studies which reported a varying, but largely acceptable or poor quality of apps for cardiological conditions such as heart failure, atrial fibrillation, or hypertension [23,24,26]. Since many MHA primarily provide information, the deficits in the average information quality are alarming [11,13]. A total of *N* = 3078 apps were identified by the web crawler and only 38 apps (3.12%) were CHD-specific and met the inclusion criteria. Above that, none of the objective subscales nor the MARS total mean scores were significantly correlated with the user star ratings which may increase the challenge of patients with CHD to identify a reliable MHA restricting the clinical use of MHA. Since none of the paid apps were among the ten highest-rated MHA, requiring payment is also not an adequate indicator of app quality. Furthermore, some MHA with very little CHD-specific information, an overwhelming amount, or questionable content were found. This included MHA implying to cure CHD only by certain yoga practices or solely by specific natural remedies. From a clinical perspective, this misleading information can have harmful effects on affected users such as not seeking professional medical advice or treatment.

Considering this, the lack of evidence regarding the usefulness and effectiveness of MHA is concerning. Only one study [37] investigating the diagnostic accuracy of one MHA could be found, which corresponds to previous app reviews, demonstrating little evidence-base for commercially available MHA [38,39,40,41]. This is in line with a validation study that included MHA for several health conditions and reported no evidence-base for 94.8% of the 1299 included MHA [34]. As many of the included MHA only consist of information texts or calculators, efficacy studies are rather inadequate, since symptom reduction due to those MHA alone is unlikely. However, studies examining for example the usability, feasibility, or user acceptance could be of importance and increase the scientific discourse on MHA. This necessity is further increased by the fact, that only few MHA (13.16%) were developed by credible sources such as universities, NGOs, or governmental organizations.

Identified functions included information, CHD risk score calculators, reminders for exercise or medication, feedback to data entries and health managers with goal setting regarding exercise, weight, and nutrition. With regard to the overall conceptualization of MHA we found that the majority comprised solely one or two functions while in only seven apps (18.42%) three or more functions were employed. The number of employed functions was positively correlated with the MARS total score, therefore those MHA with several functions were mostly among the highest-rated apps. Most MHA were limited to information and CHD risk score calculators. Thus, the majority of apps fall short of their potential to foster behavior change in patients using reminders, notifications, achievements, and encouragement and regarding important lifestyle changes like quitting smoking, being more active, or eating healthier [42]. Nevertheless, the embedment of MHA in current treatment models could most likely be valuable for patients as well as for health care providers being able to quickly access patients’ data. Independent expert rating platforms or databases such as www.mhad.science (accessed on 19 September 2021) or https://mindapps.org (accessed on 19 September 2021) are necessary to support patients and providers in finding and choosing reliable MHA of high quality [43].

This study has some limitations. First, the web crawler is limited to 200 apps per search term and the European market. This might deform the results by omitting some MHA, even though apps specifically developed for the US market were also found. Second, some apps might be locally restricted and published for specific countries only. Additionally, apps that required specific login/ access information were excluded, since they are not instantly available for most users, reducing the number of MHA. Third, only MHA in German or English language and for the chosen search terms were covered. Therefore, the number of included MHA is potentially not extensive and future studies could examine the search terms that patients use when looking for CHD apps. Fourth, the development of apps is very rapid [44], resulting in one app (‘Atherosclerosis’) no longer being available for rating by the second reviewer and some apps no longer being detectable between the first and second screening or for inclusion. Fifth, according to the standard procedure of the MARS two reviewers rated the apps, even though more raters would lead to more accurate estimates.

Sixth, with the MARS the quality of MHA is evaluated in regard to engagement, functionality, aesthetics, and information quality. In addition to those dimensions, other aspects might be relevant for app users as well and a high MARS score does not imply a high effectiveness of the app. Hence, in future studies this systematic review of MHA could be replicated with other instruments like ENLIGHT [45] or different suitable scales.

## 5. Conclusions

This first systematic evaluation of MHA for CHD demonstrated an average overall quality of MHA (*M* = 3.38, *SD* = 0.36). The most common functions were information texts and risk score calculators. Only few MHA provide a set of multiple functions and incorporate behavior change techniques limiting the potential for lifestyle changes and support in disease management of users. Most MHA were not developed by a credible source and there is a considerable lack of scientific evidence for the usefulness and efficacy of the included MHA. Nevertheless, some potentially helpful MHA were identified. The results of this study will be made publicly available to users and healthcare providers at www.mhad.science (accessed on 19 September 2021).

## Figures and Tables

**Table 1:** General characteristics of included MHA for coronary heart disease.

	*n* (%)	*M* (*SD*)
**Platform** Android iOS Both	30 (78.95%) 7 (18.42%) 1 (2.63%)	
**Affiliation** Commercial company NGO University Government Unknown	20 (52.63%) 2 (5.26%) 2 (5.26%) 1 (2.63%) 13 (34.21%)	
**Obligatory payment** Google Play store Apple App store	2 (5.26%) 2 (5.26%)	2.84 (0.85) 2.29 (1.20)
**User ratings** Google Play store Apple App store	12 (31.58%) 1 (2.63%)	4.26 (0.47) 1.0 (0.00)
**Technical aspects** Internet required App community	19 (50.0%) 1 (2.63%)	
**Methods** Information and education Tips and advice Feedback Alternative medicine Bodily exercises	34 (89.47%) 25 (65.79%) 16 (42.11%) 3 (7.89%) 2 (5.26%)	
**Security & privacy** Privacy policy Contact information Informed consent Login Password	26 (68.42%) 33 (86.84%) 7 (18.42%) 6 (15.79%) 3 (7.89%)	

Note. *n* = number of apps; *M* = mean; *SD* = standard deviation.

**Table 2:** Means of the MARS rating from highest to lowest total score.

Name	Rated on	Total Score	Quality Rating				Subjective Subscales		
			Engagement	Functionality	Aesthetics	Information Quality	E	F	G
CardiaCare	GP	4.22	4.00	4.50	4.67	3.70	2.83	3.25	2.58
Love My Heart for Women	AA	4.00	3.80	4.25	4.17	3.78	2.50	3.00	2.42
CardioVisual: Heart Health Built by Cardiologists	GP	3.84	3.30	3.88	4.17	4.00	2.67	3.00	2.08
Heart Disease Yoga & Diet–Cardiovascular disease	GP	3.83	3.70	4.13	4.00	3.50	2.83	3.13	2.50
My Heart Age	GP	3.83	3.60	4.00	4.00	3.70	2.67	3.50	2.83
ASCVD Risk Estimator Plus	GP	3.79	2.90	4.25	4.00	4.00	3.00	2.88	2.25
Texas Heart Institute	AA	3.75	2.80	4.25	4.17	3.78	2.67	2.88	2.00
The Heart App ©	GP	3.74	3.20	4.25	4.17	3.33	3.83	3.25	2.17
Angina	GP	3.59	2.90	4.13	3.83	3.50	2.67	2.63	2.08
Heart Disease 101 Audio Book	GP	3.56	2.50	4.38	4.00	3.38	2.67	3.13	2.00
Heart Disease Support	AA	3.56	3.50	4.13	3.50	3.13	2.50	2.75	1.58
Heart Diseases & Treatment	GP	3.55	2.60	4.25	3.83	3.50	2.50	2.50	1.92
MESA CHD Risk Score	GP	3.53	2.90	3.88	3.83	3.50	2.50	2.63	1.75
Healthy Heart Guides	GP	3.51	2.90	4.13	3.83	3.20	2.83	2.88	2.33
Heart Care Health & Diet Tips	GP	3.51	2.80	4.00	4.00	3.25	2.50	2.63	1.92
Basic Cardiology	GP	3.49	2.20	4.25	4.00	3.50	2.67	2.63	1.83
CardioRisk Calc	AA	3.43	2.80	4.13	3.67	3.11	2.17	2.00	1.58
Heart Disease Guide	GP	3.40	2.60	4.13	3.67	3.20	2.50	2.38	1.83
Cardiovascular Diseases	GP	3.39	2.20	4.38	3.50	3.50	2.67	2.50	2.00
Heart Disease B	GP	3.36	2.20	4.00	3.83	3.40	2.50	2.63	2.08
Cardiovascular Care Guide	GP	3.34	2.30	4.13	3.83	3.10	2.50	2.63	1.83
Heart Health Tips	GP	3.34	2.30	4.38	3.67	3.00	2.50	2.13	1.50
Atherosclerosis	GP	3.28	2.20	4.25	3.67	3.00	2.50	2.38	2.00
Heart Disease A	GP *	3.27	3.00	3.88	3.00	3.20	2.33	2.63	2.00
Heart Disease C	GP	3.26	2.50	4.13	3.33	3.10	2.67	2.63	1.92
Heart Disease Diet-Have a Fit & Healthy Heart with Best Nutrition!	AA *	3.25	2.20	4.13	3.67	3.00	2.33	1.88	1.92
Home Remedies For Chest Pain (Angina)	GP	3.24	2.00	4.25	3.83	2.88	2.00	1.75	1.67
Cardiology consultation	GP	3.21	2.80	3.88	3.17	3.00	2.33	2.13	1.83
Natural Remedies For Chest Pain (Angina)	GP	3.16	2.00	4.25	3.50	2.88	2.17	1.88	1.67
Cardiology-Expert Consult 4 Diagnosis & Treatment	GP	3.09	2.30	4.00	3.17	2.90	2.33	2.50	2.08
Angina Pectoris Disease	GP	3.08	2.20	3.75	3.50	2.88	2.67	2.50	1.75
Cardiovascular Disease Information	GP	3.08	1.90	4.25	3.17	3.00	2.50	2.00	1.75
Herz und koronarer Herzkrankhe	AA *	3.00	2.30	4.00	3.00	2.70	2.17	1.88	1.50
Arteriosclerosis Disease	GP	2.88	2.00	3.63	3.50	2.38	2.17	1.63	1.42
Heart Disease Risk Prediction and Prevention	GP *	2.87	2.60	4.00	2.50	2.38	2.17	1.75	1.42
How To Cure Heart Disease	GP	2.84	2.30	4.13	2.67	2.25	2.00	1.63	1.33
CORONARY HEART DISEASE RISK	GP	2.76	2.40	2.75	3.00	2.88	2.00	1.63	1.42
Universal Healing Programme	AA	2.50	1.80	3.25	2.67	2.30	1.67	1.63	1.33
**Total mean**	**-**	**3.38**	**2.64**	**4.06**	**3.62**	**3.18**	**2.49**	**2.45**	**1.90**

Note. * fee required. GP = Googly Play, AA = Apple App, E = Therapeutic Gain, F = Subjective Quality, G = Perceived Impact.

**Table 3:** Employed functions per included MHA for the ten highest-rated apps.

	Provision of Information					Data Acquisition, Processing and Evaluation					Calendar and Appointment-Related			Support		Other	
Name	News	Reference	Learning Material	Player/Viewer	Broker	Decision Support	Calculator	Meter	Monitor	Surveillance/Tracker	Diary	Reminder	Calendar	Utility/Aid	Coach	Health Manager	Communicator/Social Network
CardiaCare	-	✓	-	-	-	-	✓	-	-	-	-	✓	-	-	-	**✓**	-
Love My Heart for Women	-	✓	-	-	-	✓	✓	-	-	-	-	✓	✓	-	-	**✓**	-
CardioVisual: Heart Health Built by Cardiologists	-	✓	-	**✓**	-	-	✓	-	-	-	-	-	-	-	-	-	-
Heart Disease Yoga & Diet–Cardiovascular disease	-	✓	-	-	-	-	✓	-	-	-	-	✓	✓	-	-	**✓**	-
My Heart Age	-	✓	-	-	-	✓	**✓**	-	✓	-	-	-	-	-	-	-	-
ASCVD Risk Estimator Plus	-	✓	-	-	-	-	**✓**	-	-	-	-	-	-	-	-	-	-
Texas Heart Institute	-	**✓**	-	-	-	-	✓	-	-	-	-	-	-	-	-	-	-
The Heart App ©	-	**✓**	-	-	-	-	✓	-	-	-	-	-	-	-	-	-	-
Angina	-	**✓**	-	-	-	-	-	-	-	-	-	-	-	-	-	-	✓
Heart Disease 101 Audio Book	-	**✓**	-	✓	-	-	-	-	-	-	-	-	-	-	-	-	-

Note. **✓** primary function of the MHA, ✓ function is employed in the MHA.

## Data Availability

The raw data supporting the conclusions of this article will be made available by the corresponding author on request.

## Appendix A

**Table A1:** List of all search terms for coronary heart disease.

English	German
Coronary heart disease	Koronare Herzkrankheit
Coronary artery disease	Koronare Herzerkrankung
Ischemic heart disease	Ischämische Herzkrankheit
Heart disease	Ischämische Herzerkrankung
	Herzerkrankung Herzkrankheit

## Appendix B

**Table A2:** Functions of the included MHA.

	*n* (%) Android	*n* (%) iOS
**Provision of information** News Reference Learning material Player/Viewer Broker	- 31 (100%) - 5 (16.13%) -	- 6 (85.71%) - 1 (14.29%) -
**Data acquisition, processing and evaluation** Decision support Calculator Meter Monitor Surveillance/Tracker	3 (9.68%) 10 (32.26%) - 1 (3.23%) -	1 (14.29%) 3 (42.86%) - - -
**Administrative use** Administration	-	-
**Calendar and appointment-related** Diary Reminder Calendar	1 (3.23%) 2 (6.45%) 2 (6.45%)	- 1 (14.29%) 1 (14.29%)
**Support** Utility/Aid Coach Health manager	- - 2 (6.45%)	- - 1 (14.29%)
**Other** Actuator Communicator/Social network Game Store	- 1 (3.23%) - -	- 1 (14.29%) - -

## Appendix C

**Table A3:** Employed functions per included MHA.

	Provision of Information					Data Acquisition, Processing and Evaluation					Calendar and Appointment-Related			Support			Other
Name	News	Reference	Learning Material	Player/Viewer	Broker	Decision Support	Calculator	Meter	Monitor	Surveillance/Tracker	Diary	Reminder	Calendar	Utility/Aid	Coach	Health Manager	Communicator/Social Network
CardiaCare	-	✓	-	-	-	-	✓	-	-	-	-	✓	-	-	-	**✓**	-
Love My Heart for Women	-	✓	-	-	-	✓	✓	-	-	-	-	✓	✓	-	-	**✓**	-
CardioVisual: Heart Health Built by Cardiologists	-	✓	-	**✓**	-	-	✓	-	-	-	-	-	-	-	-	-	-
Heart Disease Yoga & Diet–Cardiovascular disease	-	✓	-	-	-	-	✓	-	-	-	-	✓	✓	-	-	**✓**	-
My Heart Age	-	✓	-	-	-	✓	**✓**	-	✓	-	-	-	-	-	-	-	-
ASCVD Risk Estimator Plus	-	✓	-	-	-	-	**✓**	-	-	-	-	-	-	-	-	-	-
Texas Heart Institute	-	**✓**	-	-	-	-	✓	-	-	-	-	-	-	-	-	-	-
The Heart App ©	-	**✓**	-	-	-	-	✓	-	-	-	-	-	-	-	-	-	-
Angina	-	**✓**	-	-	-	-	-	-	-	-	-	-	-	-	-	-	✓
Heart Disease 101 Audio Book	-	**✓**	-	✓	-	-	-	-	-	-	-	-	-	-	-	-	-
Heart Disease Support	-	✓	-	-	-	-	-	-	-	-	-	-	-	-	-	-	**✓**
Heart Diseases & Treatment	-	**✓**	-	-	-	-	-	-	-	-	-	-	-	-	-	-	-
MESA CHD Risk Score	-	✓	-	-	-	-	**✓**	-	-	-	-	-	-	-	-	-	-
Healthy Heart Guides	-	**✓**	-	-	-	-	✓	-	-	-	-	-	✓	-	-	-	-
Heart Care Health & Diet Tips	-	**✓**	-	-	-	-	✓	-	-	-	-	-	-	-	-	-	-
Basic Cardiology	-	**✓**	-	-	-	-	-	-	-	-	-	-	-	-	-	-	-
CardioRisk Calc	-	✓	-	-	-	-	**✓**	-	-	-	-	-	-	-	-	-	-
Heart Disease Guide	-	**✓**	-	✓	-	-	-	-	-	-	-	-	-	-	-	-	-
Cardiovascular Diseases	-	**✓**	-	-	-	-	-	-	-	-	-	-	-	-	-	-	-
Heart Disease B	-	**✓**	-	-	-	-	-	-	-	-	-	-	-	-	-	-	-
Cardiovascular Care Guide	-	**✓**	-	✓	-	-	-	-	-	-	-	-	-	-	-	-	-
Heart Health Tips	-	**✓**	-	-	-	-	-	-	-	-	-	-	-	-	-	-	-
Atherosclerosis	-	**✓**	-	-	-	-	-	-	-	-	-	-	-	-	-	-	-
Heart Disease A	-	**✓**	-	-	-	✓	-	-	-	-	-	-	-	-	-	-	-
Heart Disease C	-	**✓**	-	✓	-	-	-	-	-	-	-	-	-	-	-	-	-
Heart Disease Diet-Have a Fit & Healthy Heart with Best Nutrition!	-	**✓**	-	-	-	-	-	-	-	-	-	-	-	-	-	-	-
Home Remedies For Chest Pain (Angina)	-	**✓**	-	-	-	-	-	-	-	-	-	-	-	-	-	-	-
Cardiology consultation	-	✓	-	-	-	**✓**	-	-	-	-	-	-	-	-	-	-	-
Natural Remedies For Chest Pain (Angina)	-	**✓**	-	-	-	-	-	-	-	-	-	-	-	-	-	-	-
Cardiology -Expert Consult 4 Diagnosis & Treatment	-	**✓**	-	-	-	-	-	-	-	-	-	-	-	-	-	-	-
Angina Pectoris Disease	-	**✓**	-	-	-	-	-	-	-	-	-	-	-	-	-	-	-
Cardiovascular Disease Information	-	**✓**	-	-	-	-	-	-	-	-	-	-	-	-	-	-	-
Herz und koronarer Herzkrankhe	-	**✓**	-	-	-	-	-	-	-	-	-	-	-	-	-	-	-
Arteriosclerosis Disease	-	**✓**	-	-	-	-	-	-	-	-	-	-	-	-	-	-	-
Heart Disease Risk Prediction and Prevention	-	✓	-	-	-	-	**✓**	-	-	-	✓	-	-	-	-	-	-
How To Cure Heart Disease	-	**✓**	-	-	-	-	-	-	-	-	-	-	-	-	-	-	-
CORONARY HEART DISEASE RISK	-	**✓**	-	-	-	-	-	-	-	-	-	-	-	-	-	-	-
Universal Healing Programme	-	-	-	**✓**	-	-	-	-	-	-	-	-	-	-	-	-	-

Note. **✓** primary function of the MHA, ✓ function is employed in the MHA.
